# Frailty and its multidimensional correlates among hospitalized older adults with pulmonary tuberculosis: a cross-sectional study

**DOI:** 10.3389/fmed.2026.1923293

**Published:** 2026-08-07

**Authors:** Huijuan Wang, Meng Hao, Bofei Liu, Fangyuan Mu, Xujuan Lu

**Affiliations:** Department of Nursing, The Fourth People's Hospital of Ningxia Hui Autonomous Region, Yinchuan, China

**Keywords:** frailty, inflammation, older adults, pulmonary tuberculosis, social support

## Abstract

**Background and objective:**

Frailty is a clinically important geriatric syndrome that may increase vulnerability to adverse outcomes in older adults with chronic infectious diseases. Older adults with PTB often experience infection-related inflammation, nutritional depletion, functional decline, psychological distress, multimorbidity, and treatment burden, all of which may contribute to frailty. However, evidence regarding frailty and its associated factors in hospitalized older adults with PTB remains limited. This study aimed to assess the current status of frailty and identify multidimensional factors associated with frailty among hospitalized older adults with PTB.

**Methods:**

This hospital-based cross-sectional study consecutively enrolled older adults with PTB from two tuberculosis wards of a tertiary specialist hospital in Ningxia, China, between January and June 2026. Eligible participants were aged ≥60 years and had bacteriologically confirmed or clinically diagnosed PTB. Frailty was assessed using the FRAIL scale. Fall risk, anxiety and depressive symptoms, social support, and activities of daily living were evaluated using the MFS, HADS, SSRS, and Barthel Index, respectively. Demographic characteristics, disease-related variables, medication use, and laboratory indicators were also collected. Non-normally distributed continuous variables were described as medians and interquartile ranges. Group differences were analyzed using the Mann–Whitney *U* test or Kruskal–Wallis *H* test. Spearman correlation analysis and multiple linear regression were used to explore factors associated with FRAIL score.

**Results:**

A total of 225 questionnaires were distributed, and 219 valid questionnaires were included, yielding an effective response rate of 97.3%. The median FRAIL score was 2.00 (1.00–3.00), suggesting that many participants were in a vulnerable or pre-frail state. The median MFS, HADS-A, HADS-D, SSRS, and WBC values were 40.08 (25.00–50.00), 9.00 (7.00–11.00), 11.00 (9.00–12.00), 38.00 (34.00–42.00), and 5.30 (4.35–6.44) × 10^9^/L, respectively. Univariate analysis showed that FRAIL score differed significantly according to monthly household income per capita, sputum smear or culture result, and number of medication types. Spearman correlation analysis showed that FRAIL score was negatively correlated with MFS and HADS-A scores, but positively correlated with SSRS score and WBC count. In multivariable linear regression, WBC count, SSRS score, and MFS score remained independently associated with FRAIL score. The final model was statistically significant, with an adjusted *R*^2^ of 0.070.

**Conclusion:**

Frailty is an important clinical issue among hospitalized older adults with PTB and is associated with inflammatory status, social support, and fall-risk-related functional vulnerability. These findings suggest that frailty assessment should be integrated into routine care for older adults with PTB. Multidimensional screening that incorporates infection-related indicators, functional status, psychosocial context, and nursing care needs may help identify vulnerable patients and support individualized geriatric management during anti-TB treatment.

## Introduction

Tuberculosis (TB) remains a major global public health challenge and continues to impose a substantial disease burden despite decades of prevention and control efforts. According to the World Health Organization Global Tuberculosis Report 2025, an estimated 10.7 million people developed TB worldwide in 2024, corresponding to an incidence rate of 131 cases per 100,000 population, and approximately 1.23 million people died from TB in the same year (1). China remains one of the high TB burden countries and accounted for 6.5% of global incident TB cases in 2024 (1). Meanwhile, rapid population aging has further complicated TB control. By the end of 2024, China had 310.31 million people aged 60 years and older, accounting for 22.0% of the national population (2). National age-specific notification data from China showed that PTB notification rates were highest among people aged 65 years and older, and population aging has become an important factor slowing progress in TB control (3). Older adults are particularly vulnerable to PTB because immunosenescence, latent infection reactivation, chronic comorbidities, malnutrition, functional decline, and delayed health-seeking behavior may interact to increase disease susceptibility and worsen treatment tolerance (4).

Frailty is a multidimensional geriatric syndrome characterized by decreased physiological reserve and increased vulnerability to stressors. It is not simply equivalent to aging, disability, or comorbidity, but represents a clinically important state that predisposes older adults to falls, functional dependence, hospitalization, treatment complications, and mortality (5, 6). Several conceptual models have been widely used to assess frailty, including the physical frailty phenotype and the deficit accumulation model (6, 7). The FRAIL scale, which includes fatigue, resistance, ambulation, illnesses, and loss of weight, has also been recommended as a brief and practical screening tool for identifying frailty in clinical and community settings (8). In older adults with chronic diseases, frailty is increasingly recognized as a key indicator of biological vulnerability and resilience, and its assessment has become an important component of geriatric evaluation, chronic disease management, and risk stratification.

Older adults with PTB may be at particularly high risk of frailty because PTB combines the burden of chronic respiratory infection, systemic inflammation, nutritional depletion, reduced physical activity, psychological distress, social isolation, multimorbidity, and polypharmacy. Previous studies have shown that elderly patients with TB often have more comorbid medical conditions and different clinical presentations compared with younger patients (9). In very elderly hospitalized patients with PTB, hypoalbuminemia, respiratory failure, and dependent activities of daily living have been identified as independent risk factors for adverse drug reactions or death shortly after treatment initiation (10). A recent cohort study further demonstrated that comorbidity and multimorbidity are highly prevalent among elderly PTB patients and are associated with adverse treatment outcomes (11). These findings suggest that the prognosis of older adults with PTB is shaped not only by microbiological disease activity, but also by host vulnerability, functional reserve, nutritional status, and the broader burden of chronic disease.

Nevertheless, several important gaps remain. First, most existing studies on elderly PTB have focused on epidemiological characteristics, bacteriological findings, adverse drug reactions, or treatment outcomes, whereas frailty has received comparatively limited attention. Second, although emerging evidence suggests that frailty is common among older adults with PTB, current studies remain insufficient to clarify how infection-related factors, functional vulnerability, psychological symptoms, social support, and inflammatory or nutritional indicators are jointly associated with frailty in this population (12). Third, it remains uncertain whether routine clinical and nursing assessment tools can help identify frailty-related risk profiles among hospitalized elderly PTB patients, particularly in resource-limited specialist settings. Addressing these gaps is clinically important because early identification of frailty may support individualized nursing care, fall prevention, nutritional support, psychological intervention, medication safety, and coordinated geriatric management during anti-TB treatment.

Therefore, this cross-sectional study aimed to describe the current status of frailty among hospitalized older adults with PTB and to identify multidimensional factors associated with frailty, including demographic and disease-related characteristics, fall risk, anxiety and depressive symptoms, social support, and laboratory indicators. By integrating frailty assessment into the clinical context of PTB, this study may provide evidence for early risk screening and individualized supportive care for older adults with chronic infectious disease burden and geriatric vulnerability.

## Methods

### Study design and reporting guideline

This was a hospital-based cross-sectional study conducted among older adults hospitalized with PTB. The study was designed to assess the current status of frailty and to explore multidimensional factors associated with frailty in this population. The reporting of this study was guided by the Strengthening the Reporting of Observational Studies in Epidemiology (STROBE) statement for observational studies (13).

### Study setting and participants

Participants were consecutively recruited from two tuberculosis wards of Ningxia Fourth People’s Hospital, a tertiary specialist hospital for infectious diseases in Ningxia, China, between January and June 2026. Eligible patients were screened by trained investigators during hospitalization.

The inclusion criteria were as follows: (1) diagnosis of PTB according to the Chinese health industry standard WS 288–2017, including bacteriologically confirmed or clinically diagnosed PTB (14); and (2) age 60 years or older. Patients were excluded if they had malignant tumors or other severe diseases that could substantially interfere with frailty assessment, or if they had severe cognitive impairment, hearing impairment, speech impairment, or communication difficulties that precluded completion of the questionnaire.

### Sample size estimation

The sample size was estimated using the formula for estimating a population mean: *N* = (*Zα*/2 × *σ*/*δ*)^2^ (15). With a two-sided *α* of 0.05, *Zα*/2 of 1.96, an estimated standard deviation of 1.25 based on preliminary data, and an allowable error of 0.19, the minimum required sample size was calculated as 175. Considering a potential invalid response rate of 10%, at least 195 participants were required. In the present study, 225 questionnaires were distributed and 219 valid questionnaires were ultimately included. This final sample size not only meets the statistical requirement for single-variable population mean estimation but also satisfies the well-recognized empirical criteria for multivariate regression analysis and exploratory factor analysis (*n* > 200), which is sufficient to support the model construction and overall statistical analyses in this study.

### Variables and measurements

The primary outcome was frailty status assessed using the FRAIL scale. Potential associated factors included demographic characteristics, disease-related characteristics, functional status, fall risk, psychological symptoms, social support, and laboratory indicators.

Demographic variables included sex, age, educational level, monthly household income per capita, marital status, smoking status, alcohol consumption, and physical exercise. Disease-related variables included sputum smear or culture results, number of medications, and laboratory indicators, including white blood cell count.

### Frailty assessment

Frailty was assessed using the FRAIL scale, which consists of five components: fatigue, resistance, ambulation, illnesses, and loss of weight (8). Each item is scored as 0 or 1, with a total score ranging from 0 to 5. A score of 0 indicates robust status, 1–2 indicates pre-frailty, and ≥3 indicates frailty. In this study, the FRAIL total score was used as the main dependent variable for correlation and regression analyses, and frailty categories were used for descriptive interpretation. Higher scores indicate more severe frailty. The Cronbach’s *α* coefficient of the scale in this study was 0.702.

### Functional status

Activities of daily living were assessed using the Barthel Index (16). The scale evaluates 10 domains, including feeding, bathing, grooming, dressing, bowel control, bladder control, toilet use, transfers, mobility on level surfaces, and stair climbing. The total score ranges from 0 to 100, with higher scores indicating better functional independence. In this study, a score <60 was defined as moderate-to-severe dependence, whereas a score ≥60 was defined as mild dependence or functional independence. The Cronbach’s *α* coefficient in this study was 0.845.

### Fall risk

Fall risk was assessed using the Morse Fall Scale (17). The scale includes six items: history of falling within the past 3 months, secondary diagnosis, ambulatory aid, intravenous therapy or heparin lock, gait or transferring status, and mental status. The total score ranges from 0 to 125. A score <25 indicates low fall risk, 25–45 indicates moderate fall risk, and >45 indicates high fall risk. Higher scores indicate a greater risk of falling.

### Anxiety and depressive symptoms

Anxiety and depressive symptoms were assessed using the Hospital Anxiety and Depression Scale (18). The scale contains 14 items and consists of two subscales: HADS-A and HADS-D. Each subscale ranges from 0 to 21. Scores of 0–7 indicate normal status, 8–10 indicate mild symptoms, 11–14 indicate moderate symptoms, and 15–21 indicate severe symptoms. Higher scores indicate more severe anxiety or depressive symptoms. The Cronbach’s *α* coefficient in this study was 0.919.

### Social support

Social support was assessed using the Social Support Rating Scale developed by Xiao (19), which has been widely used in Chinese populations and includes three dimensions: objective support, subjective support, and utilization of support (20). The scale consists of 10 items covering three dimensions: objective support, subjective support, and utilization of support. The total score ranges from 12 to 66. A score ≤22 indicates low social support, 23–44 indicates moderate social support, and >44 indicates high social support. Higher scores indicate greater perceived and available social support. The Cronbach’s *α* coefficient in this study was 0.896.

### Data collection and quality control

Before the formal investigation, all investigators received standardized training from the principal investigator. The training covered the study protocol, eligibility screening, questionnaire administration, interpretation of scale items, communication procedures, and data confidentiality requirements.

During data collection, trained investigators explained the study purpose, questionnaire content, and completion requirements to each participant and, when necessary, to family members. Written informed consent was obtained before the questionnaire survey. For participants who were elderly, had poor vision, had a low educational level, or had difficulty writing, investigators read each item aloud using standardized instructions and recorded the participant’s responses according to their original answers without additional interpretation or guidance.

All questionnaires were checked immediately after completion. Missing responses, inconsistent answers, or logically contradictory information were verified with the participant in a timely manner. Questionnaires with regular-pattern responses or irreconcilable logical inconsistencies were excluded. Data were entered independently by two investigators and cross-checked to reduce data entry errors.

### Bias control

Several procedures were used to reduce potential bias. First, consecutive recruitment during the study period was used to reduce selection bias as far as possible within a single-center hospital-based design. Second, standardized eligibility criteria and diagnostic criteria were applied to ensure consistency in participant inclusion. Third, validated instruments were used to assess frailty, functional status, fall risk, psychological symptoms, and social support. Fourth, all investigators received uniform training and used standardized instructions during the survey to reduce interviewer bias. Fifth, immediate questionnaire checking and double data entry were performed to minimize missing data and information errors.

### Statistical analysis

Data were entered using Microsoft Excel and analyzed using IBM SPSS Statistics version 27.0. Categorical variables were described as frequencies and percentages. Continuous variables were first assessed for normality. Because most continuous variables were not normally distributed, they were presented as medians and interquartile ranges.

For univariate analyses, the Mann–Whitney *U* test was used to compare FRAIL scores between two groups, and the Kruskal–Wallis *H* test was used for comparisons among three or more groups. Spearman correlation analysis was used to assess the associations between FRAIL score and continuous variables, including fall risk score, anxiety score, depressive symptom score, social support score, and laboratory indicators.

Variables with statistical significance in univariate or correlation analyses, together with clinically relevant variables, were considered for multivariable analysis. Multiple linear regression was performed to identify factors independently associated with FRAIL score. Before regression modeling, multicollinearity among independent variables was evaluated using tolerance and variance inflation factor values. A tolerance value >0.10 and a variance inflation factor <10 were considered to indicate no serious multicollinearity. Regression coefficients, standard errors, standardized coefficients, *t* values, and *p* values were reported. A two-sided *p* value <0.05 was considered statistically significant.

### Ethical considerations

This study was approved by the Ethics Committee of Ningxia Fourth People’s Hospital (Ethics Approval No. 2026-QSY-014). All participants provided written informed consent before enrollment. The study was conducted in accordance with the principles of voluntary participation, confidentiality, anonymity, and data protection.

## Results

**Figure 1:**
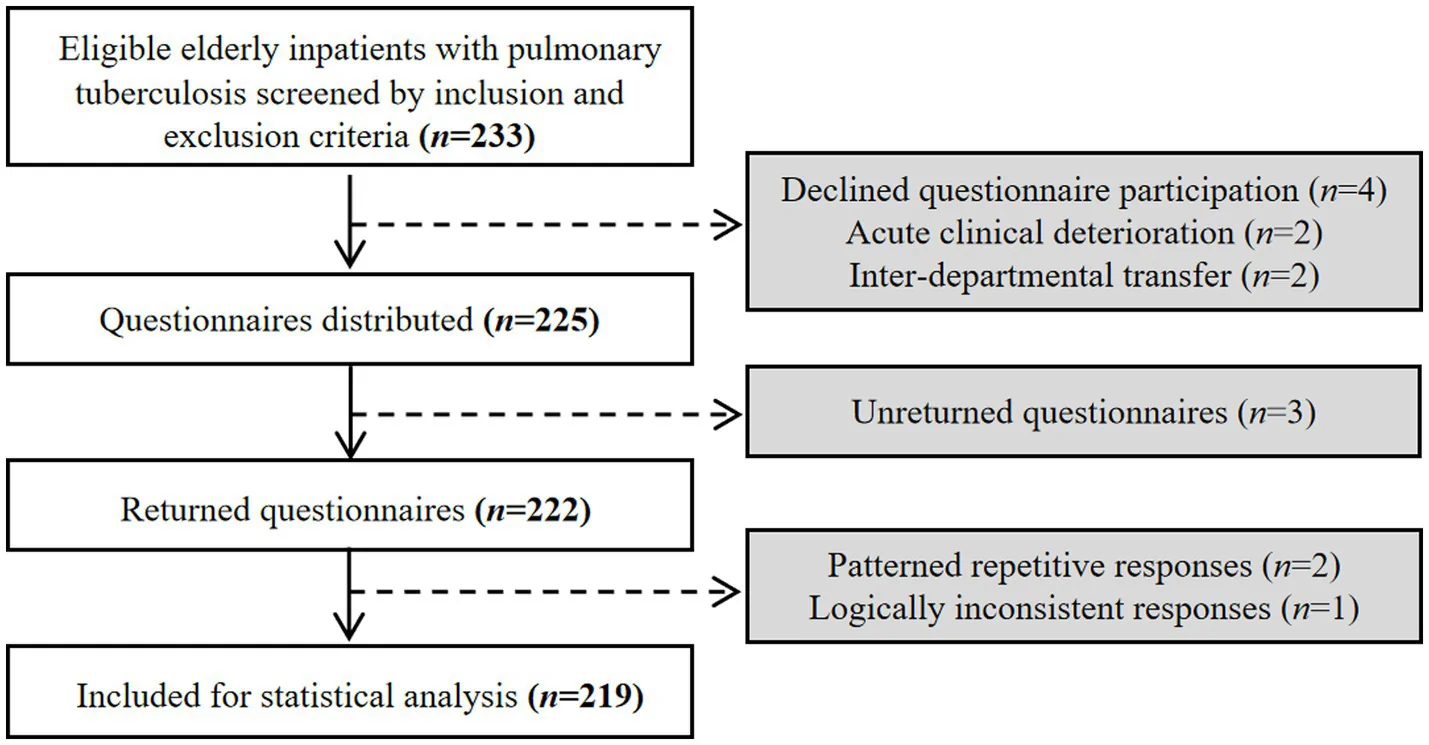
Flowchart of sample recruitment and screening.

A total of 225 questionnaires were distributed during the study period. Of these, three questionnaires were not returned. After quality screening, two questionnaires with regular-pattern responses and one questionnaire with logically inconsistent answers were excluded. Finally, 219 valid questionnaires were included in the analysis, yielding an effective response rate of 97.3% (Figure 1).

### Participant characteristics and univariate analysis of FRAIL score

Among the 219 older adults with PTB, 107 were men (48.9%) and 112 were women (51.1%). The largest age group was 71–80 years, accounting for 49.3% of the study population, followed by 60–70 years (46.1%) and >80 years (4.6%). Most participants had a primary school education or below (74.9%). Regarding bacteriological status, 174 patients (79.5%) had negative sputum smear or culture results, 36 (16.4%) had positive results, and 9 (4.1%) were not tested. Most patients had a Barthel Index score ≥60 (92.2%), and 185 patients (84.5%) were taking 0–1 type of medication.

The median FRAIL score was 2.00 across most demographic and clinical subgroups. In the univariate analysis, FRAIL scores differed significantly according to monthly household income per capita (*H* = 11.205, *p* = 0.011), sputum smear or culture result (*H* = 9.269, *p* = 0.010), and number of medication types (*H* = 7.250, *p* = 0.027). No statistically significant differences in FRAIL score were observed according to sex, age group, educational level, marital status, living arrangement, smoking status, alcohol consumption, physical exercise, Barthel Index category, or BMI category (all *p* > 0.05; Table 1).

**Table 1:** Participant characteristics and univariate analysis of FRAIL score (*n* = 219).

Characteristic	Category	*n* (%)	FRAIL score, *M* (Q1, Q3)	*Z*/*H*	*p*-value
Sex	Male	107 (48.9)	2.00 (1.00, 3.00)	−0.529	0.597
	Female	112 (51.1)	2.00 (2.00, 4.00)		
Age, years	60–70	101 (46.1)	2.00 (1.00, 3.50)	2.894	0.235
	71–80	108 (49.3)	2.00 (1.00, 3.00)		
	>80	10 (4.6)	3.00 (2.00, 4.00)		
Educational level	Primary school or below	164 (74.9)	2.00 (1.00, 4.00)	4.050	0.256
	Junior high school	16 (7.3)	2.00 (1.00, 2.00)		
	Senior high school/technical secondary school	24 (11.0)	2.00 (2.00, 3.00)		
	College or above	15 (6.8)	2.00 (1.00, 4.00)		
Monthly household income per capita, CNY	≤1,000	10 (4.6)	0.00 (0.00, 2.25)	11.205	0.011
	1,000–3,000	135 (61.6)	2.00 (2.00, 4.00)		
	3,000–5,000	42 (19.2)	2.00 (1.00, 3.00)		
	≥5,000	32 (14.6)	2.00 (1.25, 3.00)		
Marital status	Married	178 (81.3)	2.00 (1.00, 3.00)	−0.515	0.606
	Divorced/separated/widowed	41 (18.7)	2.00 (1.50, 3.50)		
Living alone	No	25 (11.4)	2.00 (0.50, 2.50)	−1.947	0.051
	Yes	194 (88.6)	2.00 (2.00, 4.00)		
Smoking	No	195 (89.0)	2.00 (1.00, 4.00)	−0.515	0.607
	Yes	24 (11.0)	2.00 (2.00, 2.75)		
Alcohol consumption	No	201 (91.8)	2.00 (1.00, 4.00)	−1.431	0.152
	Yes	18 (8.2)	2.00 (1.75, 2.00)		
Physical exercise	No	44 (20.1)	2.00 (1.25, 4.00)	−0.812	0.417
	Yes	175 (79.9)	2.00 (1.00, 3.00)		
Sputum smear/culture	Negative	174 (79.5)	2.00 (2.00, 3.25)	9.269	0.010
	Positive	36 (16.4)	2.00 (0.00, 3.00)		
	Not tested	9 (4.1)	3.00 (2.00, 4.00)		
ADL score	<60	17 (7.8)	2.00 (1.00, 3.00)	−1.197	0.231
	≥60	202 (92.2)	2.00 (2.00, 4.00)		
BMI, kg/m^2^	<18.5	34 (15.5)	2.00 (1.00, 3.25)	0.052	0.974
	18.5–23.9	93 (42.5)	2.00 (2.00, 3.00)		
	≥24.0	92 (42.0)	2.00 (1.00, 4.00)		
Number of medication types	0–1	185 (84.5)	2.00 (2.00, 4.00)	7.250	0.027
	2–3	31 (14.2)	2.00 (1.00, 3.00)		
	>3	3 (1.4)	2.00 (1.50, 2.00)		

Data are presented as *n* (%) or median (interquartile range). FRAIL scores were compared using the Mann–Whitney *U* test for two-group variables and the Kruskal–Wallis *H* test for variables with three or more groups. ADL, activities of daily living; BMI, body mass index; CNY, Chinese yuan.

### Distribution of FRAIL score and related clinical assessment scores

The median FRAIL score was 2.00 (IQR: 1.00–3.00). The median MFS score was 40.08 (IQR: 25.00–50.00), indicating an overall moderate fall risk level in this hospitalized population. The median HADS-A score was 9.00 (IQR: 7.00–11.00), and the median HADS-D score was 11.00 (IQR: 9.00–12.00), suggesting that anxiety and depressive symptoms were common among these patients. The median SSRS score was 38.00 (IQR: 34.00–42.00), indicating a moderate level of social support. The median WBC count was 5.30 (IQR: 4.35–6.44) × 10^9^/L (Table 2).

**Table 2:** Frailty-related assessment scores and Spearman correlations with FRAIL score.

Variable	Score/value, *M* (Q1, Q3)	*r*s with FRAIL score	*p*-value
FRAIL score	2.00 (1.00, 3.00)	1.000	–
MFS score	40.08 (25.00, 50.00)	−0.188	<0.01
HADS-A score	9.00 (7.00, 11.00)	−0.141	<0.05
HADS-D score	11.00 (9.00, 12.00)	0.032	>0.05
SSRS score	38.00 (34.00, 42.00)	0.173	<0.05
WBC count, ×10^9^/L	5.30 (4.35, 6.44)	0.172	<0.05

### Correlations between FRAIL score and clinical assessment scores

Spearman correlation analysis showed that FRAIL score was negatively correlated with MFS score (*r*s = −0.188, *p* < 0.01) and HADS-A score (*r*s = −0.141, *p* < 0.05). FRAIL score was not significantly correlated with HADS-D score (*r*s = 0.032, *p* > 0.05). In contrast, FRAIL score was positively correlated with SSRS score (*r*s = 0.173, *p* < 0.05) and WBC count (*r*s = 0.172, *p* < 0.05; Table 2).

### Multivariable analysis of factors associated with FRAIL score

Variables with statistical significance in univariate or correlation analyses were entered into the multivariable linear regression model according to the coding scheme shown in Supplementary Table S1. Variables that were statistically significant in the univariate or correlation analyses were entered into the multivariable linear regression model. These variables included monthly household income per capita, sputum smear or culture result, number of medication types, MFS score, HADS-A score, SSRS score, and WBC count. Multicollinearity diagnostics showed that the tolerance values ranged from 0.976 to 0.995 and the variance inflation factor values ranged from 1.005 to 1.025, indicating no serious multicollinearity among the independent variables.

The final multivariable linear regression model showed that WBC count, SSRS score, and MFS score were independently associated with FRAIL score. Higher WBC count was associated with a higher FRAIL score (*B* = 0.064, SE = 0.026, *β* = 0.161, *t* = 2.431, *p* = 0.016). Higher SSRS score was also associated with a higher FRAIL score (*B* = 0.038, SE = 0.015, *β* = 0.167, *t* = 2.521, *p* = 0.012). In contrast, higher MFS score was associated with a lower FRAIL score (*B* = −0.013, SE = 0.005, *β* = −0.178, *t* = −2.717, *p* = 0.007). The overall model was statistically significant (*F* = 6.461, *p* < 0.001), with an *R*^2^ of 0.083 and an adjusted *R*^2^ of 0.070 (Table 3).

**Table 3:** Multivariable linear regression analysis of factors associated with FRAIL score (*n* = 219).

Variable	*B*	SE	*β*	*t*	*p*-value
Constant	0.965	0.659	–	1.465	0.144
WBC count	0.064	0.026	0.161	2.431	0.016
SSRS score	0.038	0.015	0.167	2.521	0.012
MFS score	−0.013	0.005	−0.178	−2.717	0.007

## Discussion

This cross-sectional study aimed to assess the current status of frailty among hospitalized older adults with PTB and to identify multidimensional factors associated with frailty in this clinically vulnerable population. By integrating frailty assessment with fall risk, psychological symptoms, social support, and routine laboratory indicators, this study attempted to provide a more comprehensive understanding of frailty in the context of chronic infectious disease burden and geriatric vulnerability.

The main findings of this study can be summarized as follows. First, frailty and pre-frailty appeared to be common among older adults hospitalized with PTB, as reflected by a median FRAIL score of 2.00, suggesting that a considerable proportion of patients were already in a vulnerable or transitional frailty state during hospitalization. Second, univariate analysis showed that FRAIL scores differed significantly across subgroups defined by monthly household income per capita, sputum smear or culture result, and number of medication types, suggesting that socioeconomic status, disease-related characteristics, and treatment burden may be relevant to frailty in this population. Third, correlation analysis showed that FRAIL score was significantly associated with fall risk score, anxiety symptoms, social support, and WBC count, indicating that frailty in older adults with PTB may involve not only physical vulnerability, but also psychological, social, and inflammation-related dimensions. Fourth, multivariable linear regression further showed that WBC count, SSRS score, and MFS score remained independently associated with FRAIL score after adjustment for other significant variables.

A notable feature of this study is that some findings differed from the conventional clinical expectation. Higher WBC count was positively associated with higher FRAIL score, which is consistent with the assumption that systemic inflammatory burden may be linked to increased vulnerability. However, higher SSRS score was also associated with higher FRAIL score, and higher MFS score was associated with lower FRAIL score. These findings suggest that the relationships among frailty, social support, and fall risk in hospitalized older adults with PTB may be more complex than those observed in general community-dwelling older populations. In particular, greater social support may partly reflect the fact that more vulnerable patients receive more attention and assistance from family members or caregivers, rather than indicating a protective effect in cross-sectional data. Similarly, the inverse association between fall risk and frailty may be influenced by hospitalization-related factors, reduced mobility, limited exposure to fall-risk environments, or differences in how fall risk manifests in acutely ill older patients. These special findings should be interpreted cautiously but may provide important clues for understanding frailty in older adults with PTB as a condition shaped by infection burden, functional restriction, care dependency, and the hospital care environment.

Frailty has been well established as a multidimensional geriatric syndrome, but evidence specifically focusing on older adults with PTB remains limited. Previous studies have consistently shown that frailty reflects reduced physiological reserve and is associated with adverse outcomes such as falls, disability, hospitalization, treatment intolerance, and mortality in older adults (5–7). In the context of PTB, older patients have been reported to have more comorbid conditions, atypical clinical manifestations, poorer functional reserve, and a higher risk of adverse outcomes during treatment (9–11). A recent study developed a preliminary screening tool for high-risk frailty among older adults with PTB, further suggesting that frailty assessment is clinically relevant in this population (12). In addition, a newly published multicenter study protocol emphasized that frailty in older adults with TB remains underexplored and requires more systematic assessment (21). Consistent with these findings, the present study showed that the median FRAIL score was 2.00, indicating that many hospitalized older adults with PTB were in a pre-frail or frailty-prone state. This finding suggests that frailty in this population may not merely reflect chronological aging, but may result from the combined effects of chronic infection, nutritional depletion, functional restriction, multimorbidity, psychological stress, and treatment burden.

The association between inflammation and frailty has been relatively well established in geriatric research, although evidence from PTB-specific populations remains insufficient. A systematic review and meta-analysis showed that frail and pre-frail older adults had higher levels of inflammatory biomarkers, particularly CRP and IL-6, than robust individuals (22). The concept of “inflamm-aging” also suggests that chronic low-grade inflammation and impaired immune regulation are important biological backgrounds for age-related vulnerability (23). More broadly, chronic systemic inflammation has been recognized as a common pathway linking infections, physical inactivity, psychological stress, metabolic disorders, disability, and age-related chronic diseases (24). In very elderly hospitalized patients with PTB, poor nutritional reserve and functional dependence have also been associated with adverse drug reactions or death, indirectly supporting the clinical relevance of systemic vulnerability in this population (10). In the present study, WBC count was independently and positively associated with FRAIL score. Although WBC is a nonspecific inflammatory marker, this result suggests that even routine inflammatory indicators may reflect host vulnerability in hospitalized older adults with PTB. A possible mechanism is that persistent infection-related inflammatory activation may promote fatigue, appetite loss, catabolism, muscle wasting, reduced activity, and impaired recovery capacity, thereby contributing to frailty. However, because WBC is influenced by infection severity, medication, nutritional status, and other comorbid conditions, this association should be interpreted as a clinical signal rather than a causal biomarker.

The relationship between social support and frailty has been widely studied, but the direction and interpretation of this association remain context-dependent. Most studies in community-dwelling older adults have reported that stronger social support is associated with lower frailty risk; however, a recent systematic review concluded that the association between social support and physical frailty has not reached complete consensus across studies (25). Earlier work on social vulnerability also showed that social deficits and frailty are closely linked to mortality in older people, suggesting that social conditions are part of the broader vulnerability framework rather than merely external background factors (26). Social frailty studies have further suggested that reduced social participation, social isolation, and poor support utilization may contribute to functional decline and adverse outcomes in older adults (27). Contrary to the usual protective interpretation of social support, the positive correlation between SSRS score and FRAIL score identified in this study is mainly attributable to the unique disease profile and inpatient treatment context of hospitalized older adults with PTB. Patients with severe frailty demonstrate higher nursing dependency, more prominent functional limitations, and greater treatment requirements, with a significantly increased degree of physical vulnerability. Accordingly, these patients receive more intensive attention and substantial care support from family members, caregivers, and medical staff. In this cross-sectional study, the elevated social support level does not serve as a protective factor against frailty; instead, it represents a passive response and targeted intervention triggered by patients’ deteriorated frailty status. In addition, the SSRS only measures external and observable forms of social support while neglecting multiple clinically vital dimensions, including care burden, caregiver psychological strain, and the substantial caregiving resources invested by families of severely frail patients. More importantly, the high social support measured in inpatient settings primarily reflects temporary, hospital-specific emergency care and lacks long-term protective effects.

Frailty and fall risk are generally considered closely related, but their relationship may differ between community and hospitalized settings. In general older populations, frailty, impaired mobility, poor balance, psychological symptoms, medication burden, and sensory or neuromuscular deficits are recognized as important contributors to fall risk (28). A systematic review with meta-analysis showed that balance and mobility problems, medication-related factors, psychological factors, and sensory or neuromuscular factors were significantly associated with recurrent falls, and these domains can be viewed as markers of frailty (28). A recent clinical review also emphasized that fall risk assessment should consider gait, balance, prior falls, medications, cognition, vision, and environmental exposures (29). Therefore, most previous evidence would suggest a positive association between frailty and fall risk. In contrast, the present study found that higher MFS score was associated with lower FRAIL score. This seemingly inverse correlation does not negate the intrinsic causal link between physiological frailty and fall susceptibility. Essentially, frailty increases the inherent physiological susceptibility to falls in older adults. For hospitalized patients, however, severe frailty significantly limits physical mobility and leads to prolonged bed rest, greatly reducing exposure to fall-inducing scenarios. In contrast, patients with mild frailty can maintain independent ambulation, resulting in a higher probability of exposure to high-risk fall environments during hospitalization. It is worth emphasizing that increased ambulatory exposure merely indicates sufficient external conditions for falls to occur, rather than elevated physiological fall vulnerability at the individual level. Furthermore, the MFS primarily assesses fall risk based on behavioral and environmental indicators, including fall history, assistive device use, intravenous therapy status, gait function, and mental status, rather than evaluating physiological frailty severity and intrinsic fall susceptibility. Accordingly, the observed inverse correlation does not suggest a protective effect of frailty against falls. Instead, this finding demonstrates that frailty reflects stable intrinsic physiological fall vulnerability, whereas fall risk assessed by the MFS mainly represents dynamic environmental and behavioral fall exposure levels.

The associations of psychological symptoms with frailty are generally supported by previous evidence, but the findings in this study were weaker and less consistent. A systematic review and meta-analysis demonstrated a reciprocal relationship between depression and frailty in older adults: frail individuals were more likely to have depression, and individuals with depression were more likely to have frailty (30). Psychological distress may contribute to frailty through reduced appetite, sleep disturbance, inflammatory activation, decreased activity, lower treatment adherence, and impaired motivation for rehabilitation. In this study, HADS-A was weakly and negatively correlated with FRAIL score in univariate correlation analysis, whereas HADS-D was not significantly correlated with FRAIL score and neither anxiety nor depressive symptoms remained in the final multivariable model. This finding differs from much of the existing geriatric literature and should be interpreted cautiously. Possible explanations include the relatively narrow distribution of psychological symptom scores, the influence of hospitalization and acute disease stress on self-reported emotional symptoms, cultural differences in emotional expression among older patients, and the possibility that functional restriction and inflammatory burden may have stronger associations with FRAIL score than psychological symptoms in this PTB-specific inpatient setting.

Socioeconomic status, disease activity, and medication burden are recognized as relevant to frailty and poor outcomes in older adults, although their independent effects may vary across populations. A systematic review reported that socioeconomic disadvantage is generally associated with higher frailty risk, but the strength and pathway of this relationship vary according to frailty measurement, health system context, education, income, and access to care (31). Polypharmacy is also highly prevalent among frail older adults and is associated with poorer health outcomes, although the relationship may be bidirectional because frailty often coexists with multimorbidity and complex treatment needs (32). In elderly PTB patients, comorbidity and multimorbidity are common and have been associated with adverse treatment outcomes (11). Previous studies have also shown that bacteriological status and clinical presentation may differ between older and younger TB patients, reflecting differences in host response and disease manifestation (9). In the present study, monthly household income per capita, sputum smear or culture result, and number of medication types were associated with FRAIL score in univariate analysis but did not remain independent factors in the multivariable model. These results suggest that socioeconomic status, bacteriological status, and medication burden may be related to frailty at the descriptive level, Notably, the association between medication burden and frailty is not a unidirectional causal effect but a bidirectional interactive relationship:polypharmacy and complex treatment regimens can exacerbate physiological decline and frailty via adverse drug reactions and tissue damage, whereas frailty accompanied by multiple comorbidities and impaired physical function also increases clinical treatment demands and further aggravates medication burden, forming a mutual vicious cycle. Their effects may be partly mediated or confounded by functional vulnerability, inflammatory status, social support, and other clinical factors. For hospitalized older adults with PTB, frailty may therefore be better understood as the combined consequence of social disadvantage, infection-related burden, multimorbidity, treatment complexity, and reduced physiological reserve rather than as the result of any single factor.

### Limitations

This study has several limitations. First, the cross-sectional design limits causal inference. Although WBC count, SSRS score, and MFS score were independently associated with FRAIL score, the temporal sequence between these factors and frailty cannot be determined. For example, inflammatory activation may contribute to frailty, but frailer patients may also be more susceptible to infection-related inflammatory responses. Similarly, greater social support may be either a response to increased care needs or a factor influencing frailty progression. Future longitudinal studies are needed to clarify the temporal relationships among inflammation, social support, fall risk, and frailty in older adults with PTB. Second, this was a single-center study conducted in two tuberculosis wards of a tertiary specialist hospital, and participants were recruited by convenience sampling. Therefore, selection bias cannot be fully excluded. Hospitalized patients may have more severe disease, greater functional vulnerability, and higher care needs than community-dwelling or outpatient older adults with PTB. As a result, the findings may not be fully generalizable to all older adults with PTB, especially those managed in primary care, outpatient clinics, or community settings. Multicenter studies involving different hospital levels and community-based populations are needed to improve the representativeness and external validity of the findings. Third, frailty was assessed using the FRAIL scale, which is a brief and practical screening tool, but it may not fully capture the multidimensional nature of frailty. Frailty involves physical, nutritional, psychological, cognitive, social, and biological dimensions, whereas the FRAIL scale mainly focuses on fatigue, resistance, ambulation, illness burden, and weight loss. This may have led to underestimation or simplified classification of frailty in some patients. Future studies should consider combining the FRAIL scale with comprehensive geriatric assessment, physical performance tests, nutritional assessment, cognitive screening, and objective measures such as grip strength or gait speed. Fourth, several important variables that may influence frailty were not fully analyzed or were only partially included. Although laboratory indicators and chronic disease information were collected, the present analysis did not comprehensively evaluate disease severity, duration of PTB, radiological extent, nutritional intake, detailed comorbidity burden, anti-TB treatment phase, adverse drug reactions, medication adherence, sleep quality, cognitive status, or rehabilitation participation. These unmeasured or insufficiently adjusted factors may have contributed to residual confounding. Future research should establish a more detailed multidimensional dataset to better explain the mechanisms and pathways linking PTB, multimorbidity, inflammation, functional decline, and frailty. Fifth, some measurements were based on self-reported questionnaires, which may introduce recall bias and information bias. Older patients with low educational levels, visual impairment, or poor physical condition may have had difficulty understanding or completing some items, although trained investigators provided standardized assistance when necessary. In addition, psychological symptoms and social support may be influenced by hospitalization, family presence, disease stigma, and cultural factors. Future studies should use standardized interviewer training, repeated assessments, objective functional measurements, and, when possible, family or caregiver verification to improve measurement accuracy. Sixth, the regression model had a relatively low explanatory power, with an adjusted *R*^2^ of 0.070. This indicates that although the identified variables were statistically associated with FRAIL score, they explained only a small proportion of the variation in frailty. Frailty in older adults with PTB is likely influenced by multiple interacting biological, functional, psychological, social, and treatment-related factors that were not fully captured in this study. Future studies should use larger samples and more comprehensive models, such as hierarchical regression, structural equation modeling, or prediction modeling, to better characterize the complex determinants of frailty in this population. Finally, several findings, particularly the positive association between SSRS score and FRAIL score and the inverse association between MFS score and FRAIL score, should be interpreted cautiously. These associations may reflect the specific inpatient context, care dependency, limited mobility, or differences between fall-risk exposure and physiological frailty. However, potential measurement or coding issues cannot be completely ruled out. Future studies should verify these findings through data validation, repeated measurements, subgroup analyses, and prospective designs to determine whether these associations are reproducible and clinically meaningful.

## Conclusion

This study shows that frailty is an important clinical issue among hospitalized older adults with PTB and is associated with inflammatory status, social support, and fall-risk-related functional vulnerability. From a theoretical perspective, these findings support the view that frailty in older adults with chronic infectious disease burden is not a single physical decline state, but a multidimensional vulnerability phenotype shaped by infection-related inflammation, functional restriction, care dependency, psychosocial context, and treatment-related burden. This expands the understanding of frailty from general geriatric populations to a clinically specific group of older adults with PTB. From a clinical perspective, frailty assessment should be incorporated into the routine evaluation of older adults hospitalized with PTB. For individual patients, early identification of frailty may help guide personalized nursing care, fall prevention, nutritional and functional support, psychological attention, and discharge planning. For physicians and multidisciplinary teams, frailty screening may provide an additional layer of risk stratification beyond bacteriological status and routine clinical indicators, helping to identify patients who require closer monitoring and integrated geriatric management during anti-TB treatment. For the research field, these findings highlight the need for longitudinal and multicenter studies to clarify the causal pathways linking PTB, inflammation, multimorbidity, social support, functional decline, and frailty. From a social and public health perspective, the results suggest that TB control in aging populations should not focus solely on infection diagnosis and antimicrobial treatment, but should also address functional health, care needs, and geriatric vulnerability. Establishing frailty-oriented assessment and management pathways in TB specialist hospitals and community follow-up systems may help improve quality of care, reduce preventable adverse outcomes, and support healthy aging among older adults affected by chronic infectious diseases.

## Data Availability

Due to privacy protection requirements and ethical restrictions, the original dataset containing patients’ personal clinical information cannot be made publicly available. Data access can be applied by eligible researchers via the institutional ethics committee. Requests to access the datasets should be directed to Huijuan Wang, 1478597646@qq.com.
