# Supplementary material for: Frailty and its multidimensional correlates among hospitalized older adults with pulmonary tuberculosis: a cross-sectional study
Source: Front Med (Lausanne). 2026 Aug 7;13:1923293. doi: 10.3389/fmed.2026.1923293 (PMC13493224; doi:10.3389/fmed.2026.1923293)
Supplement: Supplementary file 1 [file Table_1.docx]

**Supplementary Table S1. Assignment of independent variables included in the multivariable model**

| **Independent variable** | **Assignment method** |
| --- | --- |
| Monthly household income per capita | <=1,000 CNY = 1; 1,000-3,000 CNY = 2; 3,000-5,000 CNY = 3; >=5,000 CNY = 4 |
| Sputum smear/culture | Negative as reference; positive = dummy variable 1 (1, 0); not tested = dummy variable 2 (0, 1) |
| Number of medication types | 0-1 = 1; 2-3 = 2; >3 = 3 |
| MFS score | Actual measured value |
| HADS-A score | Actual measured value |
| SSRS score | Actual measured value |
| WBC count, ×10⁹/L | Actual measured value |
